# Pulmonary arterial hypertension in idiopathic inflammatory myopathies: Data from the French pulmonary hypertension registry and review of the literature

**DOI:** 10.1097/MD.0000000000004911

**Published:** 2016-09-30

**Authors:** Sébastien Sanges, Cécile M. Yelnik, Olivier Sitbon, Olivier Benveniste, Kuberaka Mariampillai, Mathilde Phillips-Houlbracq, Christophe Pison, Christophe Deligny, Jocelyn Inamo, Vincent Cottin, Luc Mouthon, David Launay, Marc Lambert, Pierre-Yves Hatron, Laurence Rottat, Marc Humbert, Eric Hachulla

**Affiliations:** a University of Lille, INSERM U995, LIRIC, Lille Inflammation Research International Center; b CHU Lille, Département de Médecine Interne et Immunologie Clinique; c Centre National de Référence Maladies Systémiques et Auto-immunes Rares (Sclérodermie Systémique), Lille; d University Paris-Sud, Faculté de Médecine, Université Paris-Saclay; e AP-HP, Service de Pneumologie, DHU Thorax Innovation, Hôpital Bicêtre, Le Kremlin-Bicêtre; f INSERM UMR_S999, LabEx LERMIT, Centre Chirurgical Marie-Lannelongue, Le Plessis-Robinson; g Département de Médecine Interne et Immunologie Clinique, Centre National de Référence Maladies Neuromusculaires, Hôpital La Pitié-Salpêtrière, AP-HP, INSERM U974, Université Paris VI Pierre et Marie Curie, Paris; h Clinique Universitaire de Pneumologie, Centre Hospitalier Universitaire, Grenoble, France; i Université Joseph Fourier, Grenoble; j Service de médecine interne et rhumatologie 3C/5D, Centre Hospitalier Universitaire Pierre Zobda-Quitman; k Département de Cardiologie, Centre Hospitalier Universitaire Pierre Zobda-Quitman, Fort-de-France, Martinique; l Hospices Civils de Lyon, Service de Pneumologie, Centre de Compétence de l’Hypertension Pulmonaire, Centre de Référence des Maladies Pulmonaires Rares, Lyon; m Service de Médecine Interne, Centre de Référence des Vascularites Nécrosantes et de la Sclérodermie Systémique, Université Paris Descartes, Hôpital Cochin, Paris, France.

**Keywords:** antisynthetase syndrome, connective tissue diseases, dermatomyositis, inclusion body myositis, myositis, polymyositis, pulmonary hypertension

## Abstract

Occurrence of pulmonary arterial hypertension (PAH) in idiopathic inflammatory myopathies (IIMs) without extensive interstitial lung disease (ILD) has rarely been described in the medical literature. This study aimed to report all cases with association of PAH and IIM in the French Pulmonary Hypertension (PH) Registry, to identify IIM features associated with the presence of PAH, and to describe treatment modalities of these patients.

All cases of IIM-PAH were retrieved from the French PH Registry, which gathers PH patients prospectively enrolled by 27 referral hospital centers across France. Patients were excluded if they had an extensive ILD or overlap syndrome. Characteristics of IIM-PAH patients were compared with a control group of IIM patients without PH.

Among the 5223 PH patients in the Registry, 34 had a diagnosis of IIM. Among them, 3 IIM-PAH patients (2 females and 1 male) had no evidence of extensive ILD or overlap syndrome, and were included in this study. In these 3 patients, dermatomyositis (DM) was the only identified IIM. One patient had autoantibodies classically associated with IIM (anti-Ku). PAH had always developed after IIM onset, was severe in all cases, and led to a marked functional impairment.

By pooling our cases with 6 patients previously reported in the literature, and comparing them with a control cohort of 35 IIM patients without PH, we identify several IIM characteristics possibly associated with PAH occurrence, including DM subtype (78% vs 46%; *P* = 0.02), skin involvement (*P* = 0.04), anti-SSA antibodies (*P* = 0.05), and peripheral microangiopathy (*P* = 0.06).

Overall, IIM-PAH patients were managed by corticosteroids and/or immunosuppressants, either alone or combined with PAH therapy. Patients did not seem to respond to IIM treatment alone.

Our study reports for the first time the rare but possible association of PAH and IIM in a large prospective PH Registry. In that setting, PAH seems associated with DM, skin involvement, peripheral microangiopathy, and anti-SSA positivity. The best therapeutic strategy for IIM-PAH remains to be defined.

## Abstract 2

Supplemental Digital Content is available in the text

## 1 Introduction

Pulmonary arterial hypertension (PAH) is a rare condition characterized by a proliferation and remodeling of the small pulmonary arteries, leading to a progressive increase in pulmonary vascular resistance and right heart failure.^[1]^ Categorized as group 1 in the pulmonary hypertension (PH) classification, PAH can be idiopathic, heritable, and associated with drug exposure or with an underlying disease.^[2]^

Connective tissue diseases (CTDs) are the most frequent associated causes of PAH.^[1]^ Among them, PAH is a well-known complication of systemic sclerosis (SSc)^[3]^, systemic lupus erythematosus (SLE),^[4]^ and mixed connective tissue disease (MCTD).^[5]^ Although more scarce, the occurrence of PAH has also been documented in primary Sjögren syndrome (SjS)^[6]^ and antiphospholipid syndrome.^[7]^

Idiopathic inflammatory myopathies (IIMs) are a group of disorders classified within the CTD and characterized by an immune-mediated muscle injury.^[8]^ These disorders include mainly dermatomyositis (DM), polymyositis (PM), and inclusion-body myositis (IBM).^[8]^ Occurrence of PH due to chronic respiratory diseases (group 3 PH) has been well-documented in the context of IIM associated with antisynthetase syndrome (ASS), in a recent work by our group.^[9]^ Conversely, the association of PAH and IIM without extensive ILD has rarely been reported so far^[10–15]^; and in most cases, other causes of pulmonary hypertension (notably overlap syndromes with another CTD; and group 3 PH) could not be formally excluded.

Using data from the French PH prospective Registry, we conducted a nationwide search for cases and report here the first cohort of fully characterized IIM-PAH patients.

## 2 Methods

### 2.1 Inclusion and exclusion criteria

Eligible patients were identified through screening of the French PH Registry, which gathers all PAH cases prospectively enrolled by 27 referral hospital centers across France between 2002 and 2015 (as previously described^[1]^).

They were included in the study if they fulfilled all the following criteria: a diagnosis of PAH according to the 2015 European Society of Cardiology (ESC)/European Respiratory Society (ERS) guidelines,^[2]^ defined by a mean pulmonary arterial pressure (mPAP) ≥25 mm Hg, a pulmonary vascular resistance (PVR) ≥3 Wood units (WU), and a pulmonary artery wedge pressure (PAWP) ≤15 mm Hg, measured during a resting right-heart catheterization (RHC); a definite diagnosis of IIM, according to Dalakas criteria^[16]^ (for PM and DM), or Griggs criteria^[17]^ (for IBM); an age above 18 years old.

Patients were excluded if they met one of the following criteria: an overlap syndrome with another CTD (SSc, SLE, MCTD); an extensive ILD, defined by an extent of lung parenchymal involvement >20% on high-resolution computed tomography (HRCT) of the chest and/or a forced vital capacity (FVC) <70% of the predicted value on pulmonary function tests (PFTs); another plausible cause of PH (heritable mutation, drug or toxic exposure, HIV infection, portal hypertension, congenital cardiomyopathy, left heart disease, chronic lung disease, chronic thromboembolic PH).

The study was approved by local ethic committees and complied with the requirements of the “Commission Nationale de l’Informatique et des Libertés,” in accordance with current French legislation. This study followed the recommendations of the Helsinki Declaration of 1975, as revised in 1983.

### 2.2 Data collection for IIM-PAH patients

Regarding PH, data were recorded prospectively. Patients underwent a comprehensive evaluation, including clinical assessment, New York Heart Association (NYHA) functional class scoring, non-encouraged 6-minute walking test (6MWT), resting RHC with acute vasoreactivity testing, resting PFT, HRCT of the chest, ventilation/perfusion (V/Q) lung scan, arterial blood gases in room air, transthoracic echocardiography (TTE), and serum brain natriuretic peptide (BNP) levels. PAH treatments were recorded in the Registry. A positive response to vasoreactivity testing was defined as a reduction of mPAP ≥10 mm Hg to reach an absolute value of mPAP ≤40 mm Hg, with an increased or unchanged cardiac output (CO).^[2]^

Regarding IIM, data were retrospectively retrieved from medical records and comprised a clinical evaluation (muscle, joint, skin, and microvascular involvements), biological data (creatinine phosphokinase [CPK] and C-reactive protein [CRP] levels), immunological profile (antinuclear antibodies [ANAs], anti-double stranded DNA [anti-dsDNA], antiextractable nuclear antigen [anti-ENA], IIM-specific or associated autoantibodies, SSc-associated autoantibodies), electromyographic testing (EMG), muscle biopsy, and ongoing specific treatments. Patients were considered to have peripheral microvascular involvement if they had one of the following signs: Raynaud phenomenon, telangiectasia, digital ulcer, abnormal nailfold capillaroscopy. IIM-specific or associated autoantibodies included anti-synthetase (anti-Jo1, anti-PL7, anti-PL12, anti-EJ, anti-OJ), anti-Mi2, anti-SRP, anti-Ku, anti-PM-Scl, anti-TIF1, anti-MDA5 (CADM140), anti-NXP2 (MJ), and anti-SAE1 antibodies. SSc-associated autoantibodies included anticentromere, antitopoisomerase I, anti-RNA polymerase III, and anti-U1-RNP antibodies.

### 2.3 Constitution of a control cohort

To study statistical associations of IIM characteristics with PAH occurrence, a control cohort was retrospectively designed and recruited from all consecutive patients referred to our Department in the Lille University Hospital Center between 2002 and 2015. They were included in the cohort if they fulfilled the same criteria as described above (ie, a definite diagnosis of IIM, an age above 18 years old, no overlap syndrome, no extensive ILD, and no condition associated with PH) and if they displayed no sign suggestive of pulmonary hypertension on TTE.

### 2.4 Statistical analysis

Data are expressed as number (percentage), mean (standard deviation), or median (interquartile range). The associations of IIM characteristics with PAH occurrence were evaluated using Fisher exact or Mann–Whitney tests. No statistical comparison was done for dichotomous variables with less than 3 patients per group. A 2-tailed *P* < 0.05 was considered statistically significant. Statistical analyses were performed using SPSS software, package 22.

## 3 Results

**Figure 1:**
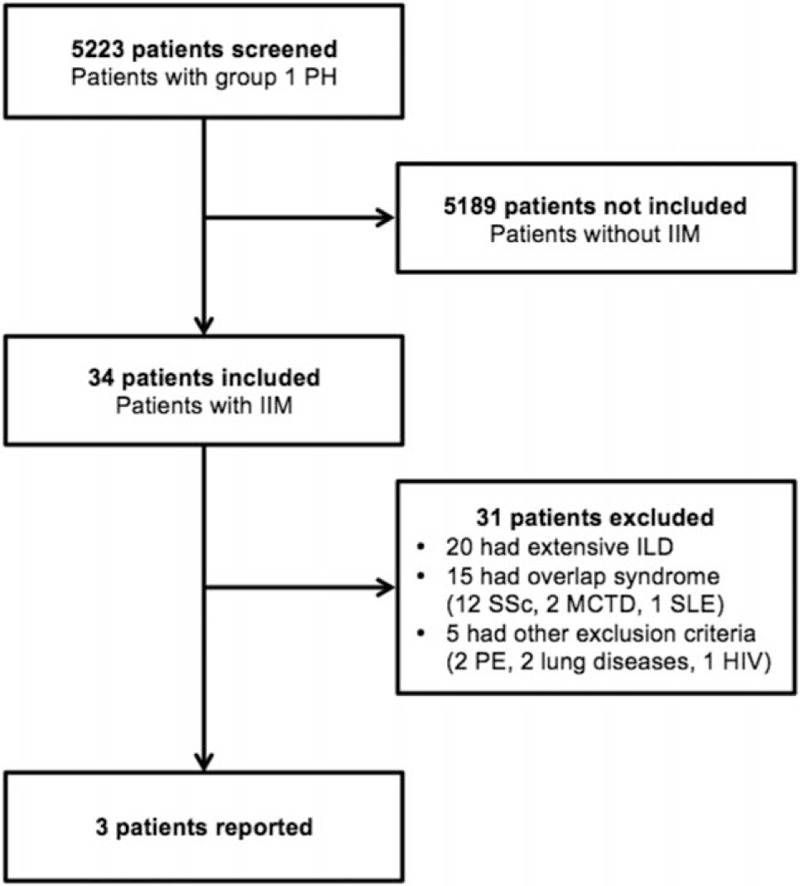
Flowchart of the study population. HIV = human immunodeficiency virus, IIM = idiopathic inflammatory myopathies, ILD = interstitial lung disease, MCTD = mixed connective tissue disease, PE = pulmonary embolism, PH = pulmonary hypertension, SLE = systemic lupus erythematosus, SSc = systemic sclerosis.

Among the 5223 PH patients registered in the Registry, 34 had a diagnosis of IIM. Among them, 31 met an exclusion criterion, mostly because of an extensive ILD and/or an overlap syndrome (Fig. 1). Three patients with a definite diagnosis of IIM-PAH could therefore be included in our study: a 66-year-old Caucasian female (patient #1), a 33-year-old Afro-Caribbean female (patient #2), and a 31-year-old Caucasian male (patient #3). A comprehensive description of their baseline characteristics is detailed in Table 1 ; and their complete medical history is available as Supplementary data.

**Table 1:**
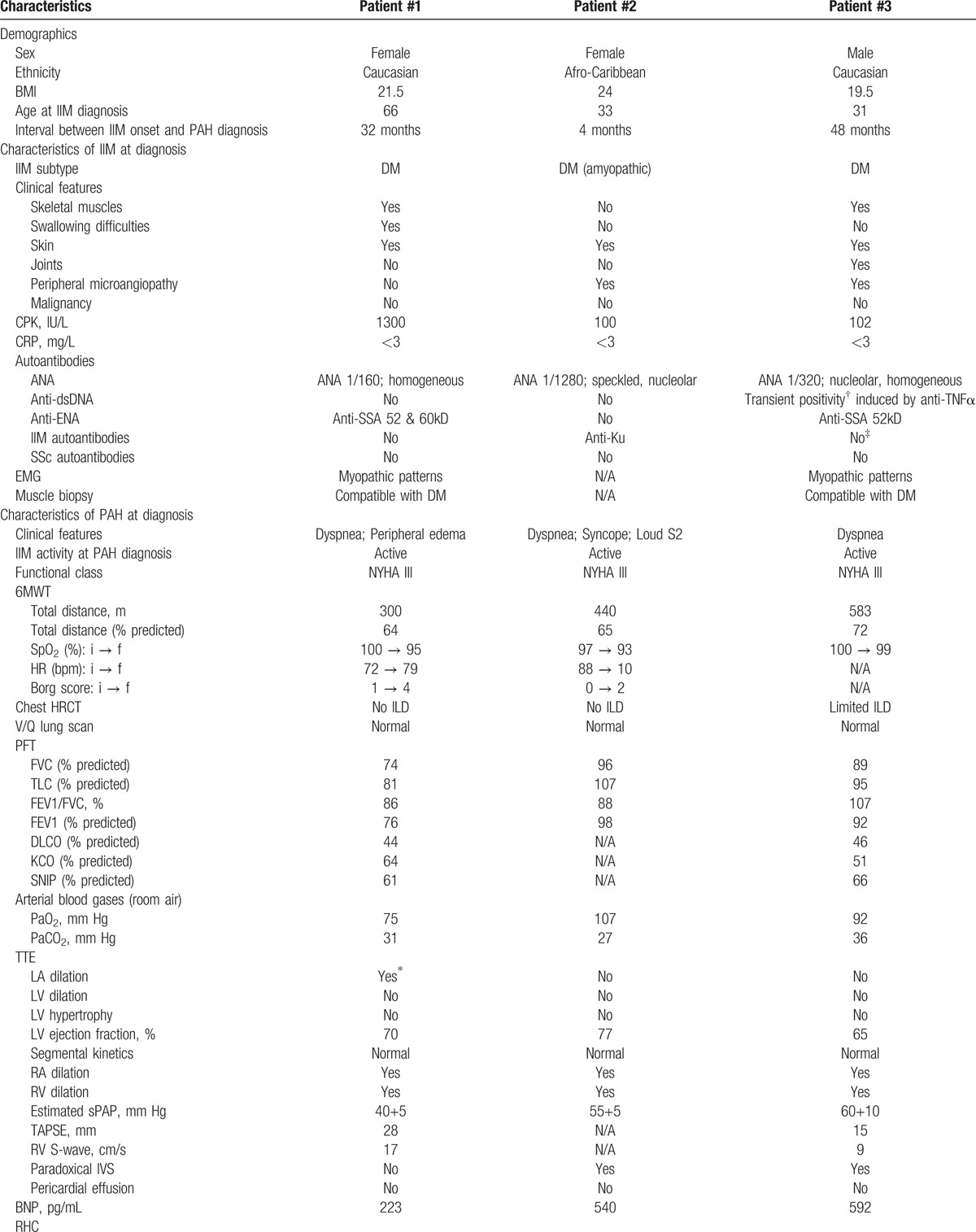
Baseline characteristics of the study population.

**Table 1 (Continued):**
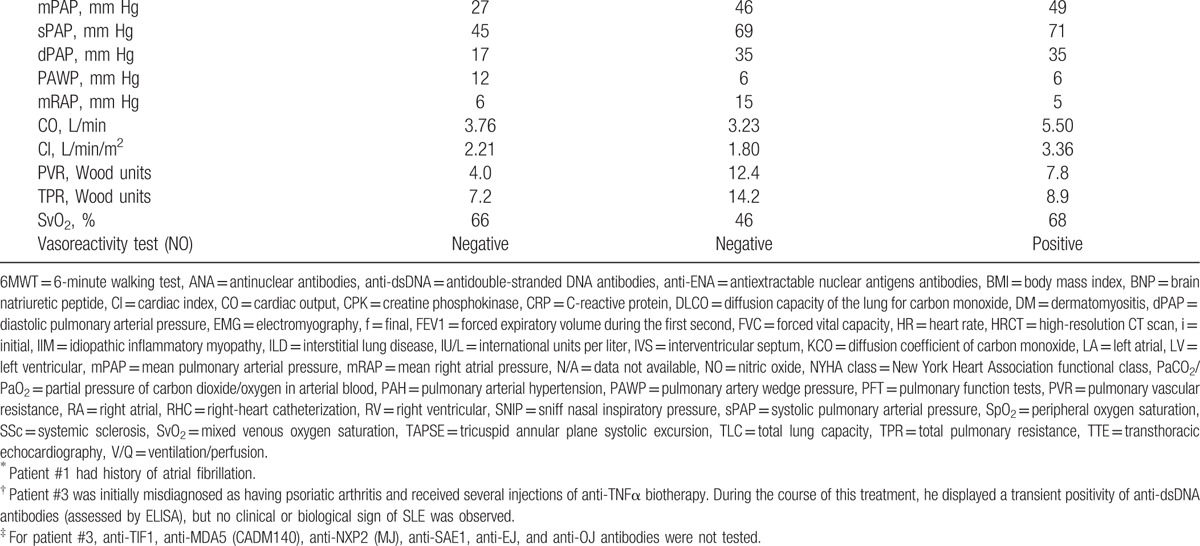
Baseline characteristics of the study population.

### 3.1 IIM characteristics in IIM-PAH patients

**Figure 2:**
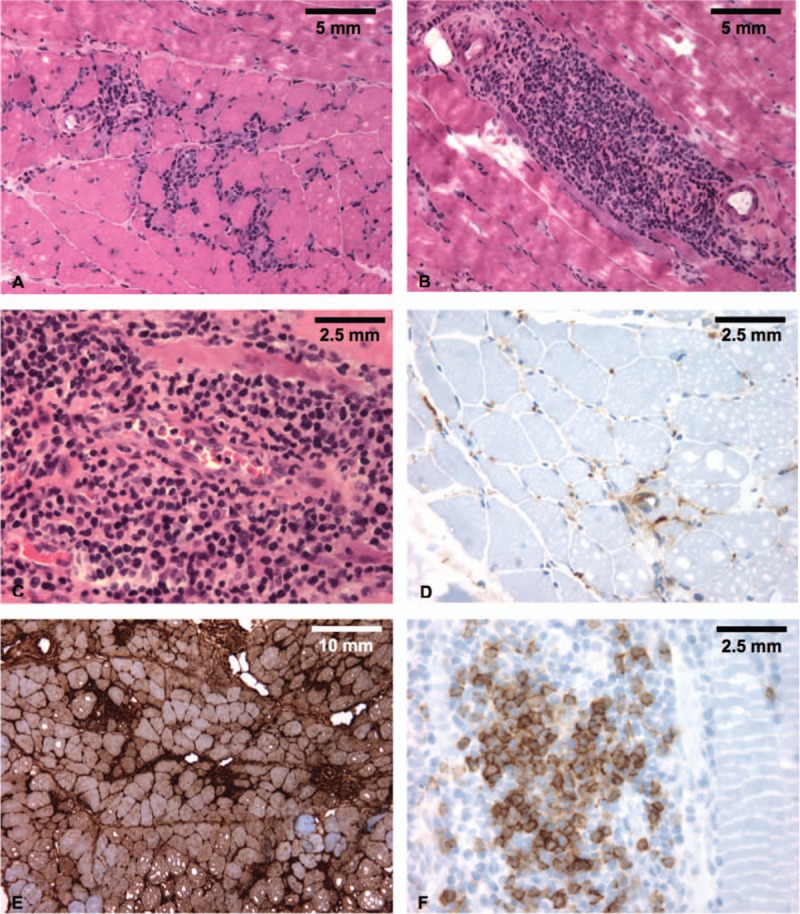
Muscle biopsy of patient #1. Representative images of patient #1's muscle biopsy, performed at the time of IIM diagnosis, showing histological features compatible with dermatomyositis. A, B, C, Hematoxylin-Erythrosin-Saffron (HES) staining (A, B: 20×; C: 40×), showing endomysial (A) and perivascular (B, C) inflammation, with mild perifascicular atrophy (A). D, Terminal complement membrane attack complex (C5b9) staining (40×), showing mild capillary C5b9 deposition. E, Major histocompatibility complex type 1 (MHC 1) staining (10×), showing diffuse membrane positivity. F, CD3 staining (40×), showing predominant T-cell inflammation (Claude-Alain Maurage, Université de Lille, F-59000 Lille, France).

**Figure 3:**
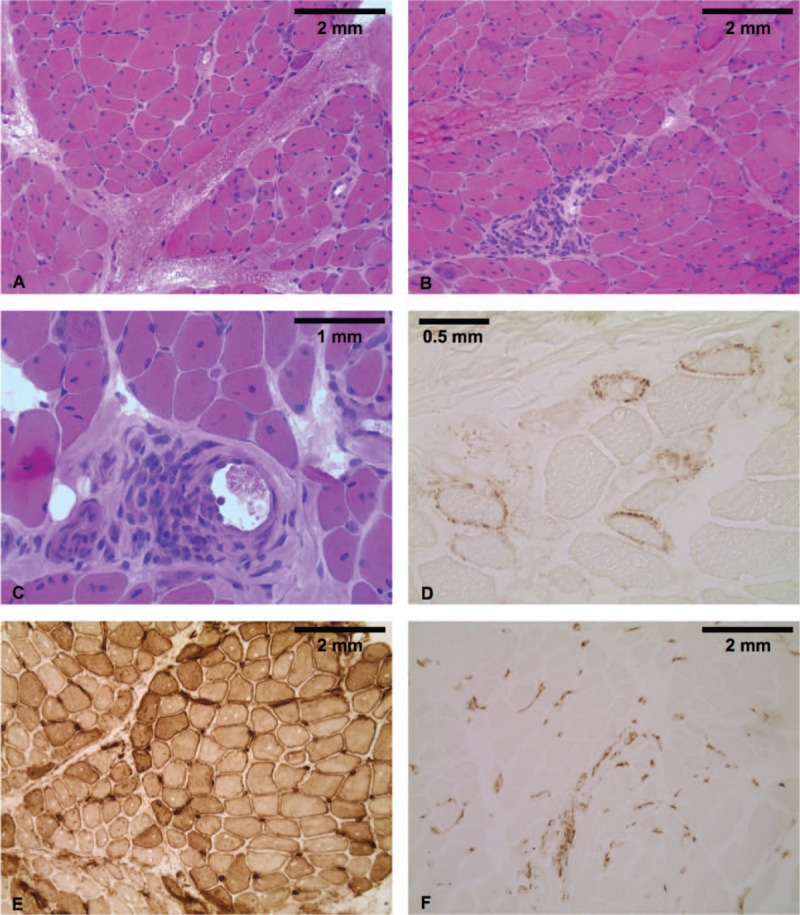
Muscle biopsy of patient #3. Representative images of patient #3's muscle biopsy, performed at the time of IIM diagnosis, showing histological features typical of dermatomyositis. A, B, C, Hematoxylin-Eosin-Safran (HES) staining (A, B: 20×; C: 40×), showing perifascicular atrophic fibers (A), perivascular and perimysial inflammation (B, C), and nuclear internalisations (B). D, Terminal complement membrane attack complex (C5b9) staining (63×), showing membrane deposition around several fibers. E, Major histocompatibility complex type 1 (MHC 1) staining (20×), showing diffuse membrane positivity with perifascicular enhancement. F, CD4 staining (20×), showing perivascular and endomysial CD4+ T-cell infiltrates (Dr Diane Giovannini, Département d’Anatomie et de Cytologie Pathologiques-IBP, CHU de Grenoble, France).

Dermatomyositis was the only IIM identified in our 3 patients. Patients #1 and #3 had a definite diagnosis of myopathic DM according to Dalakas criteria; and patient #2 was diagnosed with an amyopathic form of the disease. Typical skin features were found in all patients: Gottron papules (2/3), heliotrope rash (2/3), psoriasiform plaques (3/3), and manicure sign (1/3). Signs of peripheral microangiopathy were present in patients #2 (giant capillaries) and #3 (Raynaud phenomenon and dystrophic capillaries); nailfold capillaroscopy was normal in patient #1. Muscle involvement was variable: inexistent in patient #2, moderate (muscle pain without weakness) in patient #3, and severe (muscle weakness, swallowing difficulties and increased CPK levels) in patient #1. In myopathic patients, muscle involvement was confirmed by EMG and muscle biopsy (Figs. 2 and 3).

Antinuclear antibodies were positive in all 3 patients. Patient #2 was positive for anti-Ku antibodies, but displayed no sign of SSc. In patients #1 and #3, no IIM-specific or associated antibody was identified; anti-SSA antibodies were mildly positive, but none of them exhibited signs of SjS or SLE (Table 1 ).

Therapeutic management of DM included corticosteroids (3/3), either alone (1/3) or in combination with azathioprine (2/3) and/or hydroxychloroquine (1/3). Skin and muscle involvements improved in patients #2 and #3; however, patient #1 needed monthly injection of intravenous immunoglobulins to control the disease. After 3 to 8 years of follow-up, none of them developed any features of overlap syndrome (Table 2).

**Table 2:**
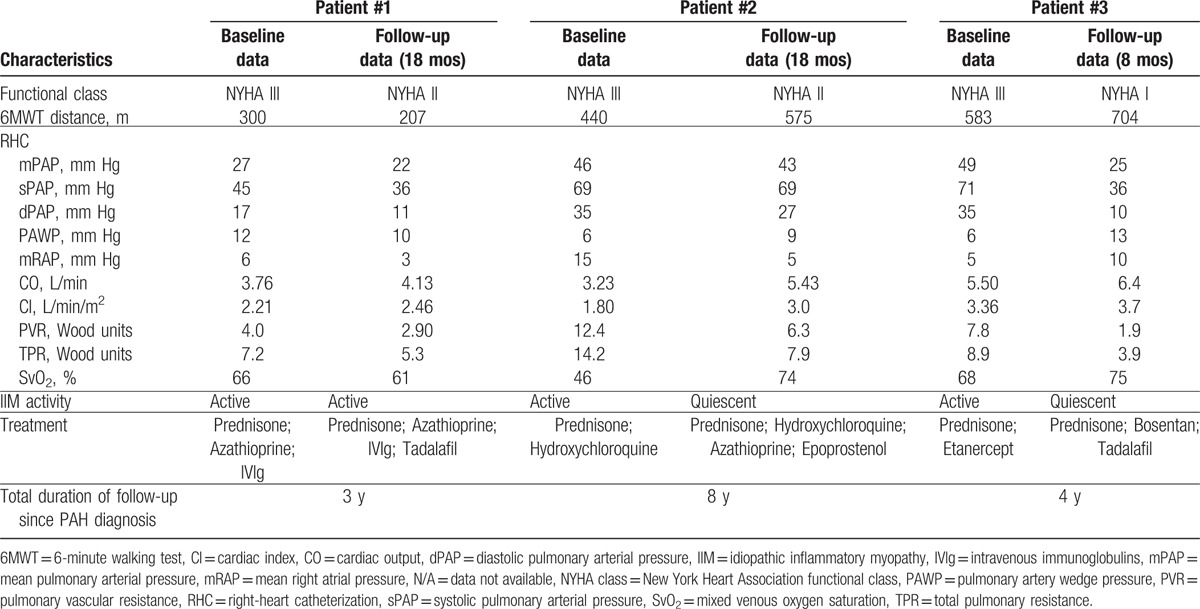
Follow-up characteristics of the study population.

### 3.2 PAH characteristics in IIM-PAH patients

Pulmonary arterial hypertension always developed after IIM onset. All patients were referred for severe dyspnea, associated with syncope and/or clinical signs of right-heart failure, which developed while their DM was still active. Precapillary PH was diagnosed by RHC, as recommended by guidelines,^[2]^ in all cases (patient #1: mPAP 27 mm Hg, PVR 4.0 WU; patient #2: mPAP 46 mm Hg, PVR 12.4 WU; patient #3: mPAP 49 mm Hg, PVR 7.8  WU) (Table 1 ). All of them had a severe PAH and functional impairment (NYHA class III; 6MWT distance between 64% and 72% of predicted value).

**Figure 4:**
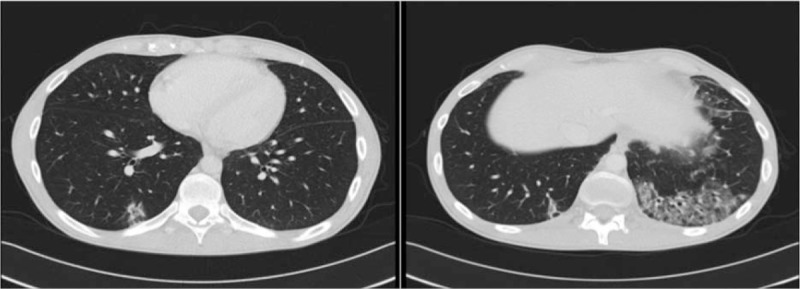
Chest HRCT of patient #3. Representative images of patient #3's thoracic HRCT at the time of PAH diagnosis, showing ground glass opacities mainly located in the left inferior lobe, and occupying less than 20% of total lung parenchyma. HRCT = high-resolution computed tomography, PAH = pulmonary arterial hypertension.

In each case, other causes of dyspnea and differential diagnoses of PH were excluded: RHC demonstrated precapillary PH, and TTE showed no sign of myocarditis or left heart failure; V/Q lung scan and helical CT of the chest excluded thromboembolic pulmonary disease; chest HRCT did not show evidence of extensive ILD (only patient #3 presented a limited ILD, as illustrated in Fig. 4); PFT displayed no obstructive or restrictive pattern.

All patients were started on PAH therapy (Table 2). In patient #1, oral tadalafil was introduced and allowed a rapid improvement of dyspnea and hemodynamic parameters. However, 6MWT distance decreased during follow-up, probably because IIM remained active. Patient #2 was treated with intravenous epoprostenol, which led to an increase of cardiac index and functional capacity. Patient #3 had a positive acute vasodilator response with inhaled NO and was started on nifedipine. As this treatment rapidly failed, it was switched to bosentan after 1 month. Three months later, tadalafil was added to bosentan, because of insufficient response to monotherapy. This sequential combination therapy allowed functional and hemodynamic improvements. All patients remained stable during the next years of follow-up (Table 2).

### 3.3 Identification of IIM characteristics associated with PAH occurrence

To determine whether certain IIM characteristics were associated with PAH occurrence, our 3 original observations were pooled with 6 previously reported cases^[10–15]^ (Table 3) and compared with a control cohort of 35 IIM patients without PH. Other reports were identified,^[18–22]^ but were not included in the analysis because of insufficient patient information. Characteristics of IIM patients with PAH and without PH are described in Table 4.

**Table 3:**
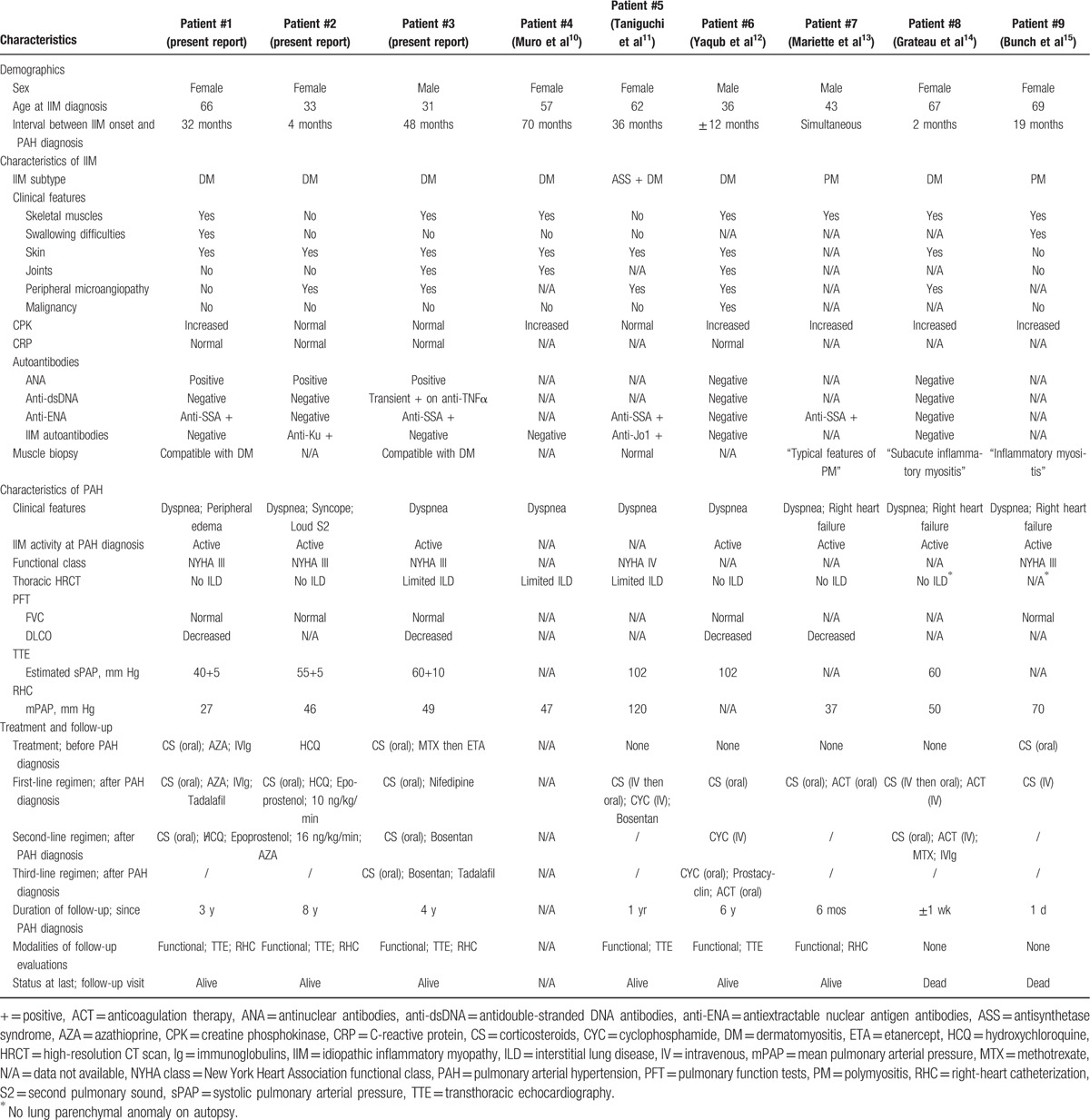
Individual data for the IIM-PAH cohort (study population and previously reported cases).

**Table 4:**
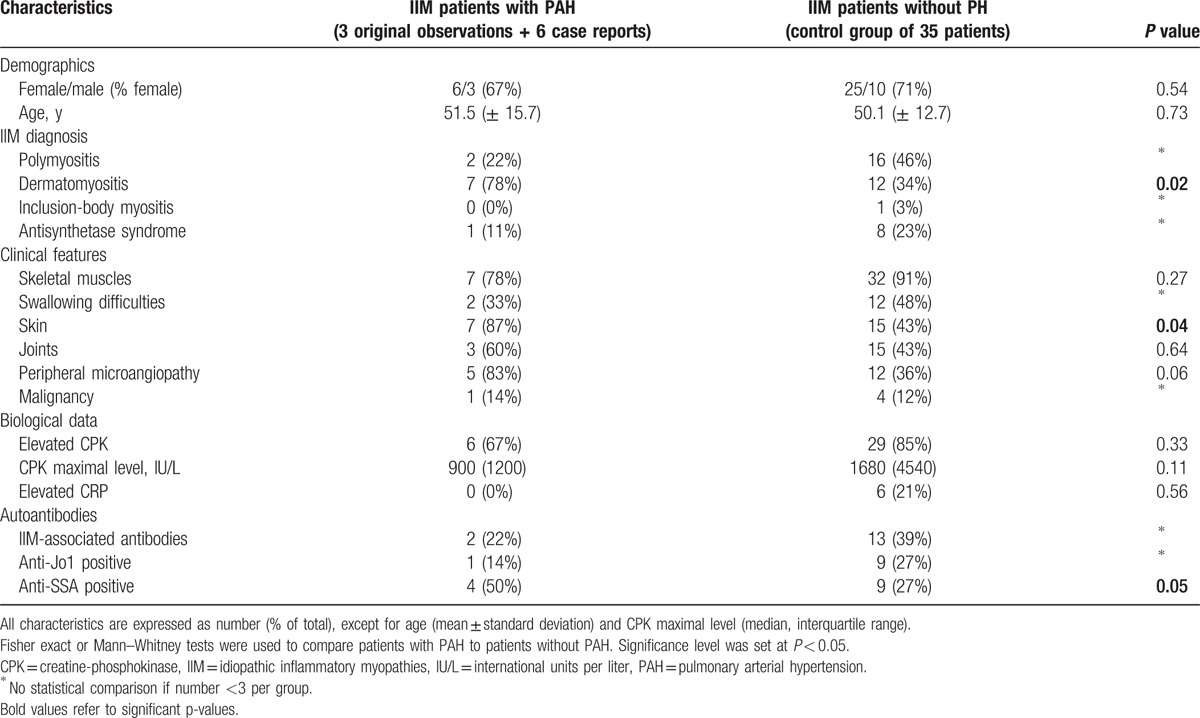
Characteristics of IIM patients with and without PAH.

Sex ratio and age at diagnosis were similar between the 2 groups. Remarkably, a diagnosis of DM was significantly associated with PAH occurrence: 78% of the patients with PAH had DM compared with 46% of the non-PH patients (*P* < 0.05). As such, presence of skin involvement was also associated with PAH (87% vs 43%; *P* < 0.05). Muscle features were comparable between the 2 cohorts, but IIM-PAH patients tended to have lower CPK levels (*P* = 0.11). Interestingly, a trend for an association between PAH and peripheral vascular disorders was found (83% vs 36%; *P* = 0.06). Finally, anti-SSA antibodies, but not IIM-specific autoantibodies, were a more frequent finding in PAH patients (50% vs 15%; *P* = 0.05).

### 3.4 Therapeutic modalities in IIM-PAH patients

Among the 9 identified IIM-PAH cases, treatment data are available in 8 patients (Table 3). Four of them (patients #1, #2, #3, and #9) were under therapy for IIM at the time of PAH occurrence, mostly by corticosteroids and/or immunosuppressants; and the 4 others were treatment-naïve.

After PAH diagnosis, 3 patients (#1, #2, and #5) were started on a mixed regimen combining PAH-specific treatments with corticosteroids and/or immunosuppressants: a clinical, echocardiographic, and/or hemodynamic improvement was observed in all of them (Table 3). The 5 remaining patients were managed by introduction or intensification of IIM treatment only: with 1 notable exception (patient #7), this strategy was associated with a deterioration of functional, TTE, and/or RHC parameters (patients #3, #6, #8, and #9). A PAH-specific treatment was then introduced in 2 patients (patient #3 after 1 month; patient #6 after 12 months) and led to an overall improvement. The last 2 patients (# 8 and #9) did not receive any PAH therapy and died rapidly.

## 4 Discussion

To our knowledge, this is the first study describing prevalent cases of IIM-PAH patients in a nationwide prospective PH registry.^[23]^ Our results can be summarized as follows: PAH is a very rare, but possible complication of IIM; among IIM characteristics, DM subtype, skin involvement, peripheral microangiopathy, and anti-SSA antibodies might be associated with PAH occurrence; IIM treatment alone might not be sufficient to stabilize PAH.

Our study benefited from a national recruitment of patients and a prospective collection of PAH data. Interestingly, only 3 patients out of 5223 prevalent PH cases were identified. This result confirms the empirical impression that, conversely to other CTDs,^[1]^ occurrence of PAH during the course of IIM is an exceptional event. Considering that PAH and IIM are rare conditions, a coincidental association, although possible, seems unlikely.

Both IIM and PAH were carefully characterized, thus ensuring that other causes of PH were effectively excluded (mostly, overlap with SSc and chronic lung diseases). Patient #2 was positive for anti-Ku antibodies, but as she displayed no sign of SSc during an 8-year follow-up, the possibility of an overlap syndrome was deemed unlikely. Interestingly, although more frequent in the context of SSc-IIM overlaps, anti-Ku antibodies have also been described in patients with isolated IIM^[24]^ and PAH.^[25]^ Cases of PAH in IIM patients have been seldom reported so far.^[10–15,18–22]^ In most published cases, phenotyping of IIM and/or PAH was incomplete, either lacking RHC data,^[12,18]^ detailed PFT results,^[10,11,13,18]^ immunological profile,^[15,18,21,22]^ exhaustive histological work-up,^[15,18,21,22]^ or sufficient follow-up.^[13–15,21,22]^ Even though IIM-PAH remains the most plausible cause of PAH in these previously published cases, PH associated with ILD or overlap syndrome with another CTD was not formally ruled out.

So far, PH in the context of IIM has been mainly described in patients with extensive ILD.^[9,18,19,26–33]^ Recently, our team identified 16 cases of hemodynamically-proven PH among 203 consecutive patients presenting with ASS, a condition characterized by the presence of anti-tRNA synthetase antibodies and associated with IIM and ILD.^[9]^ Almost all of them had extensive ILD according to Goh criteria,^[34]^ with marked limitation of functional capacities (NYHA functional class II–III; mean 6MWT distance ± standard deviation: 59% ± 19% of predicted value). The occurrence of PH considerably worsened the prognosis, with a 3-year survival rate of 58%. Similarly, Minai^[26]^ reported a series of 3 PM-DM patients who developed severe PH during the course of an ILD. All of them had a major restrictive lung disease (with a FVC ranging between 36% and 58%) and severe functional impairment (NYHA class IV, 6MWT distance between 65 and 346 m). Despite initiation of off-label PAH therapy, 2 patients died after a 12 and 21-month follow-up, respectively.

Interestingly, our report indicates that IIM-PAH patients were more likely to have a DM diagnosis (a condition whose prime pathophysiological target is thought to be endothelial cells, and not muscle fibers),^[8]^ skin manifestations, and possibly signs of peripheral microangiopathy. This suggests that IIM-PAH may be the result of a specific microvascular disease, as observed in the muscles and skin of DM patients. Indeed, in an early autopsy series of IIM, histological features of pulmonary vasculitis were found in 5 out of 65 patients,^[35]^ one of which had been previously diagnosed with PAH.^[15]^ Remarkably, the pathological aspects of the microvascular inflammation (active necrotizing or chronic proliferative vasculitis, with lymphomononuclear and plasmacytic infiltrates) were very close to those encountered during SSc and SLE.^[35]^ Similarly, Grateau et al^[14]^ reported the case of an IIM-PAH patient who died from right heart failure: post mortem histologic examination revealed pathological features that resembles idiopathic PAH (thickening, fibrosis, and massive hyalinization of the wall of small pulmonary arterioles). More recently, we noted that most patients with ILD-PH in ASS had severe hemodynamic alterations in regard to their lung parenchymal involvement,^[9]^ and we speculated that this might be due to an underlying microvascular disease.^[36]^ The occurrence of PAH in IIM without extensive ILD, as demonstrated in our present study, tends to support this hypothesis and suggests a possible benefit of PAH-specific therapy.^[37]^

Given the few number of identified cases, defining the best therapeutic strategy for IIM-PAH is challenging. However, by carefully analyzing patient data under treatment, it seems that 2 distinct trends can be identified: either patients were treated with a mixed regimen (combining IIM and PAH therapy) and seemed to stabilize or improve (patients #1, #2, and #5); either they were treated by IIM therapy alone and seemed to deteriorate (patients #3, #6, #8, and #9). This observation tends to suggest that, conversely to PAH associated with SLE and MCTD,^[38]^ IIM-PAH might not respond to corticosteroids and/or immunosuppressants alone, whereas PAH-specific therapy appeared to stabilize the disease.

Our study has several limitations. As all clinicians are not familiar with the possibility of PAH occurring during IIM, and since other causes of dyspnea are frequent in these diseases, those patients might have been underdiagnosed. Moreover, our statistical analysis should be interpreted with caution, as it could be biased by the low number of patients in each group and by the retrospective collection of IIM data.

In conclusion, our study suggests that PAH is a rare but possible complication of IIM. It should be considered in case of unexplained dyspnea, syncope, or right heart failure, especially in patients with DM subtype, skin involvement, peripheral microangiopathy, and anti-SSA antibodies. The pathogenesis of IIM-PAH is unclear and might involve a specific microvascular disease. The best therapeutic modalities for these patients remain to be defined.

## Acknowledgments

The authors wish to thank Professor Claude-Alain Maurage (Lille) and Dr Diane Giovannini (Grenoble) for their pathological expertise.

The authors also acknowledge the members of the French Pulmonary Hypertension Registry that contributed to this study: Patrice Poubeau (Saint-Pierre), François Picard (Bordeaux), Laurent Têtu (Toulouse), Arnaud Bourdin (Montpellier), Elisabeth Diot, Patrice Diot (Tours), Fabrice Bauer (Rouen).

## Supplementary Material

Supplemental Digital Content
