# Supplementary material for: Pulmonary arterial hypertension in idiopathic inflammatory myopathies: Data from the French pulmonary hypertension registry and review of the literature
Source: Medicine (Baltimore). 2016 Sep 30;95(39):e4911. doi: 10.1097/MD.0000000000004911 (PMC5265921; doi:10.1097/MD.0000000000004911)
Supplement: Supplemental Digital Content [file medi-95-e4911-s001.doc]

**Supplementary data. Detailed description of IIM-PAH patients from this study**

*Patient #1*

A 69-year-old Caucasian woman was referred to our center for a rapidly progressive onset of dyspnea, classified as NYHA functional class III.

She was diagnosed with typical DM 3 years before. At the time, she displayed both characteristic cutaneous features (Gottron papules, periorbital heliotrope rash and psoriasiform plaques on the elbow) and severe muscle involvement (marked proximal muscle weakness in upper and lower limbs, swallowing difficulties). She denied any clinical sign of peripheral microvascular disease (Raynaud phenomenon, digital ulcer or telangiectasia); and nailfold capilaroscopy was normal. Laboratory work-up revealed elevated CPK levels at 1300 IU/l. EMG showed typical myopathic patterns. Immunological testing was positive for antinuclear antibodies, with mild positivity for anti-SSA 50 & 60kD antibodies. No other autoantibody specificity was identified. Finally, a muscle biopsy displayed pathological features compatible with inflammatory myopathy (*Figure 2*). She was diagnosed with DM and started on corticosteroid in combination with azathioprine. Monthly injections of intravenous immunoglobulins were subsequently needed to control the disease.

At the time of referral, the patient exhibited signs of right cardiac failure with lower limbs edema. Muscle and skin involvements were still active, but unchanged. Pulmonary auscultation found no inspiratory crackles.

V/Q lung scan and HRCT of the chest showed no evidence of pulmonary embolism or ILD. PFT revealed an isolated decrease of DLCO, without obstructive or restrictive pattern. TTE found no evidence of left heart failure but an elevated estimated sPAP at 45 mmHg. RHC found mildly elevated values of mPAP (22 mmHg) and a low cardiac index (2.2 L/min/m^2^). After a few months of follow-up without clinical improvement, hemodynamic parameters were controlled demonstrating pre-capillary PH (mPAP 27 mmHg, PAWP 12 mmHg, CI 2.21 L/min/m^2^, PVR 4.0 Wood units). Acute vasoreactivity test was negative.

A treatment by a phosphodiesterase type 5 inhibitor (tadalafil 40 mg per day) was initiated, leading to an improvement of functional class (NYHA II) and hemodynamic parameters. 6MWT distance remained impaired though, probably due to a persistent activity of her DM. She is currently stable with treatment in her third year of follow-up.

*Patient #2*

A 33-year-old Afro-Caribbean woman was referred for an acute-onset NYHA functional class III dyspnea and syncope that occurred during her 29^th^ week of pregnancy.

A few months before, she presented with multiple skin manifestations (Gottron papule, psoriasiform plaques of the elbow, manicure sign) suggestive of DM. She had a history of preeclampsia without thromboembolic event. She denied any sign of muscle involvement (no weakness, myalgia, or increase of CPK levels). As such, EMG and muscle biopsy were not performed. Careful examination of the nailfold bed revealed giant capillaries. Immunological work-up was found positive for antinuclear antibodies, with anti-Ku specificity. She displayed no sign suggestive of systemic sclerosis (notably, no skin fibrosis). She was thus diagnosed with an amyopathic form of DM and initially started on hydroxychloroquine.

When referred, physical examination revealed a persistence of cutaneous features without muscle involvement, and no sign of deep vein thrombosis or heart failure. A loud S2 was heard on cardiac auscultation; and no inspiratory crackles were found on pulmonary auscultation. An echocardiography was quickly performed and demonstrated a major elevation of sPAP at 60 mmHg, as well as no argument for a myocarditis or left heart failure. Pulmonary embolism or interstitial lung disease were excluded by V/Q lung scan and HRCT of the chest. RHC was then performed demonstrating pre-capillary PH (mPAP 46 mmHg, CI 1.80 L/min/m^2^, PVR 12.4 Wood units), non-responsive to acute vasoreactivity testing.

Given the unfavorable prognosis of PAH during pregnancy, she was immediately treated with an intravenous prostacyclin analog (epoprostenol 10 ng/kg/min) and high-dose oral corticosteroids (prednisone 1 mg/kg/day). RHC parameters were controlled after 5 weeks of treatment, right before delivery, and were markedly improved (mPAP 29 mmHg, CI 4.10 L/min/m^2^). She delivered a healthy baby by caesarean section during her 34^th^ week of pregnancy.

However, 6 months after delivery, a worsening of pulmonary hemodynamics (mPAP 42 mmHg, CI 2.42 L/min/m^2^) was observed during a control RHC. Therapy was therefore adjusted, with a majoration of epoprostenol doses (up to 16 ng/kg/min) and the addition of azathioprine (100 mg/d).

With this treatment, a clinical, functional and hemodynamical improvement was noted and persisted during the next 8 years of follow-up. She did not develop any feature of overlap syndrome (notably with SSc) during that time.

*Patient #3*

A 35-year-old man was referred for progressive onset of dyspnea (NYHA functional class III).

Four years before, he presented with disseminated psoriasic plaques and inflammatory arthralgias of proximal interphalangeal joints and knees. Antinuclear antibodies were found positive with no specificity. He was therefore diagnosed with psoriasis and started on methotrexate and corticosteroids. Two years later, as cutaneous signs persisted, methotrexate was substituted by anti-TNFα biotherapy.

At the time of referral, the patient complained of muscle pain without weakness, shortness of breath, weight loss and fatigue. Careful anamnesis revealed that the patient had suffered from episodes of periorbital heliotrope rash and Raynaud phenomenon during the last 4 years. Physical examination also noted signs of calcinosis cutis. Nailfold capillaroscopy revealed dystrophic and enlarged capillaries, without giant capillaries. Serum CPK levels were normal, but EMG found typical myopathic patterns. A deltoid muscle biopsy supported the diagnosis of DM by showing characteristic histological anomalies.

Of note, immunological investigations were repeated at the time of referral and found a positivity of anti-dsDNA antibodies (assessed by ELISA). As the patient never displayed any clinical or biological signs of SLE, this result was interpreted as being either a false positive (due to the lack of specificity of ELISA testing) or an anti-TNFα-induced immunological anomaly with no clinical consequence. He also had a mild positivity of anti-SSA 52kD antibodies.

An extensive work-up was then performed to explain the dyspnea. No pulmonary embolism or ILD was identified. PFT revealed a decreased DLCO at 46% of the predicted value, without restrictive or obstructive pattern. TTE showed an elevated estimated sPAP (70 mmHg), associated with a dilation of right heart chambers. RHC confirmed the diagnosis of severe pre-capillary PH with a preserved CI (mPAP 49 mmHg, CI 3.36 L/min/m^2^, PVR 7.8 Wood units). There was a positive response to NO vasoreactivity challenge (mPAP 38 mmHg, CI 4.0 L/min/m^2^, PVR 4.8 Wood units).

Calcium-channel blockers (nifedipine) and high-dose oral corticosteroids (prednisone 1mg/kg/d) were introduced. After a one-month follow-up, a clinical and echocardiographic evaluation showed a deterioration of functional and TTE parameters. Nifedipine therapy was stopped and an endothelin receptor antagonist therapy (bosentan) was initiated. Three months later, clinical and hemodynamic response to bosentan was insufficient and sequential combination therapy with tadalafil was proposed. This resulted in an improvement of functional and hemodynamic parameters. The patient is currently stable on combination therapy with bosentan and tadalafil in his fourth year of follow-up. He did not develop any feature of overlap syndrome (notably with SLE) during that time.
